# Pack-Year Smoking History: An Inadequate and Biased Measure to Determine Lung Cancer Screening Eligibility

**DOI:** 10.1200/JCO.23.01780

**Published:** 2024-03-27

**Authors:** Alexandra L. Potter, Nuo N. Xu, Priyanka Senthil, Deepti Srinivasan, Hang Lee, G. Scott Gazelle, Lydia Chelala, Wei Zheng, Florian J. Fintelmann, Lecia V. Sequist, Jessica Donington, Julie R. Palmer, Chi-Fu Jeffrey Yang

**Affiliations:** ^1^ Division of Thoracic Surgery, Department of Surgery, Massachusetts General Hospital, Boston, MA; ^2^ Slone Epidemiology Center at Boston University, Boston, MA; ^3^ Biostatistics Center, Massachusetts General Hospital, Boston, MA; ^4^ Department of Radiology, Massachusetts General Hospital, Boston, MA; ^5^ Institute for Technology Assessment, Massachusetts General Hospital, Boston, MA; ^6^ Department of Radiology, University of Chicago Pritzker School of Medicine, Chicago, IL; ^7^ Division of Epidemiology, Department of Medicine, Vanderbilt Epidemiology Center, Nashville, TN; ^8^ Vanderbilt-Ingram Cancer Center, Vanderbilt University Medical Center, Nashville, TN; ^9^ Mass General Cancer Center, Massachusetts General Hospital, Boston, MA; ^10^ Section of Thoracic Surgery, Department of Surgery, University of Chicago Hospital, Chicago, IL

## Abstract

**PURPOSE:**

Pack-year smoking history is an imperfect and biased measure of cumulative tobacco exposure. The use of pack-year smoking history to determine lung cancer screening eligibility in the current US Preventive Services Task Force (USPSTF) guideline may unintentionally exclude many high-risk individuals, especially those from racial and ethnic minority groups. It is unclear whether using a smoking duration cutoff instead of a smoking pack-year cutoff would improve the selection of individuals for screening.

**METHODS:**

We analyzed 49,703 individuals with a smoking history from the Southern Community Cohort Study (SCCS) and 22,126 individuals with a smoking history from the Black Women's Health Study (BWHS) to assess eligibility for screening under the USPSTF guideline versus a proposed guideline that replaces the ≥20-pack-year criterion with a ≥20-year smoking duration criterion.

**Results:**

Under the USPSTF guideline, only 57.6% of Black patients with lung cancer in the SCCS would have qualified for screening, whereas a significantly higher percentage of White patients with lung cancer (74.0%) would have qualified (*P* < .001). Under the proposed guideline, the percentage of Black and White patients with lung cancer who would have qualified for screening increased to 85.3% and 82.0%, respectively, eradicating the disparity in screening eligibility between the groups. In the BWHS, using a 20-year smoking duration cutoff instead of a 20-pack-year cutoff increased the percentage of Black women with lung cancer who would have qualified for screening from 42.5% to 63.8%.

**Conclusion:**

Use of a 20-year smoking duration cutoff instead of a 20-pack-year cutoff greatly increases the proportion of patients with lung cancer who would qualify for screening and eliminates the racial disparity in screening eligibility between Black versus White individuals; smoking duration has the added benefit of being easier to calculate and being a more precise assessment of smoking exposure compared with pack-year smoking history.

**Figure:**
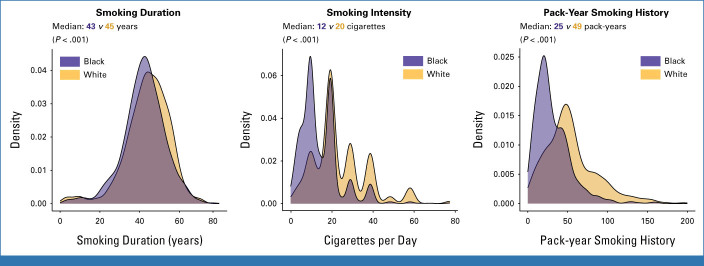

## INTRODUCTION

Early detection of lung cancer through low-dose computed tomography (LDCT) screening is one of the most promising strategies to reduce lung cancer mortality among high-risk individuals.^1,2^ LDCT screening is recommended by the US Preventive Services Task Force (USPSTF) for individuals meeting certain age and smoking history requirements.^3^ However, many individuals at high risk for lung cancer, especially those from racial and ethnic minority groups, are ineligible because they have too few pack-years of smoking.^4-11^

**CONTEXT**

**Key Objective**
Does the use of a smoking duration cutoff instead of a pack-year cutoff to determine lung cancer screening eligibility improve the selection of individuals for screening?
**Knowledge Generated**
In this analysis of two large cohort studies, use of a 20-pack-year smoking history cutoff as a selection criterion for lung cancer screening excluded many individuals diagnosed with lung cancer and led to a marked racial disparity in screening eligibility between Black versus White individuals. Revising the US Preventive Services Task Force guideline to include a 20-year smoking duration cutoff (instead of a 20-pack-year smoking history cutoff) increased the proportion of patients with lung cancer who would qualify for screening and eliminated the racial disparity in screening eligibility.
**Relevance *(I. Cheng)***
This epidemiological study provides evidence supporting the use of smoking duration as an alternative criterion to pack-years in determining eligibility for lung cancer screening; this strategy may reduce the racial disparities in lung cancer screening between Black and White individuals.*
  *Relevance section written by *JCO* Associate Editor Iona Cheng, PhD, MPH.

Pack-year smoking history—a composite measure based on smoking intensity and smoking duration—is a widely accepted clinical tool to quantify an individual's tobacco exposure and assess their risk of lung cancer.^12,13^ However, the use of pack-year smoking history to determine lung cancer screening eligibility is based on historic precedent, emerging from its use as an eligibility criterion in the National Lung Screening Trial (NLST).^2^ To date, there have been no studies comparing the use of pack-year smoking history versus other measures of tobacco exposure to select individuals for lung cancer screening, and there are presently no data to support the use of smoking pack-years (as opposed to other measures of tobacco exposure) in determining lung cancer screening eligibility.

Of note, the use of pack-years as a measure of cumulative tobacco exposure has been previously criticized on the grounds that it incorrectly assumes that smoking intensity and smoking duration have equal importance in determining lung cancer risk (ie, they are given equal weight in the calculation of a pack-year).^12,19,20^ Importantly, studies have shown that smoking duration is more strongly associated with lung cancer risk than smoking intensity.^21-24^ Thus, the use of a pack-year smoking history cutoff to select individuals for screening may exclude many individuals at high risk for lung cancer by underestimating lung cancer risk among those who smoke less intensely (ie, fewer cigarettes per day), such as individuals from racial and ethnic minority groups.^25-27^

The current USPSTF guideline recommends lung cancer screening for individuals age 50-80 years with at least a 20-pack-year smoking history and who either currently smoke or have quit smoking within the past 15 years.^3^ The objective of this study was to evaluate the impact of a proposed guideline that uses smoking duration instead of pack-years as a criterion for lung cancer screening. We hypothesize that this simple change to the USPSTF guideline would increase the proportion of individuals diagnosed with lung cancer who would have qualified for screening and reduce the disparity in screening eligibility between Black and White individuals. We chose to focus on the disparity in screening eligibility between Black versus White individuals because (1) Black individuals face a disproportionate burden of lung cancer in the United States, with Black individuals more likely to be diagnosed with advanced-stage lung cancer^28,29^ and to have worse survival compared with White individuals,^28,30^ and (2) there is a large body of evidence supporting a marked disparity in screening eligibility between Black versus White individuals.^4-11^

## METHODS

### Study Design

The study was conducted among Black and White individuals from the Southern Community Cohort Study (SCCS) and Black women from the Black Women's Health Study (BWHS; see the Data Supplement, online only for details). Informed consent was obtained from each individual who enrolled in the SCCS and BWHS, respectively. The Institutional Review Boards (IRB) at Vanderbilt University and Meharry Medical College approved the SCCS, and the IRB at Boston University approved the BWHS. Study participants who had never smoked (see the Data Supplement for details), had unknown or missing smoking information, or had a history of lung cancer at study enrollment were excluded. Study participants' race was ascertained through self-report. Incident lung cancers (International Classification of Disease for Oncology, Third Edition^31^ codes C340-C349) were identified via linkage with state cancer registries and the National Death Index up to December 31, 2021, for both studies. Smoking characteristics were obtained from baseline and follow-up questionnaires, with active follow-up every 5 years in the SCCS and every 2 years in the BWHS (see the Data Supplement for details). Smoking data were missing for only 4% of SCCS and 0.5% of BWHS participants (Data Supplement, Table S1).

Primary analyses were conducted in data from the SCCS to enable comparisons between Black and White individuals. Confirmatory analyses were conducted in BWHS data.

### Statistical Analysis

Sensitivity and specificity of the 2021 USPSTF guideline versus proposed guideline were calculated for each study cohort. Sensitivity and specificity using different smoking duration thresholds (eg, ≥10 years, ≥30 years) were also evaluated. In addition, the proportion of individuals with a smoking history (with or without lung cancer) in each study cohort who would have qualified for screening under the 2021 USPSTF guideline versus proposed guideline was calculated. Differences in the sensitivity and specificity of the 2021 USPSTF guideline versus proposed guideline and differences in the proportion of individuals eligible under the 2021 USPSTF guideline versus proposed guideline were assessed using McNemar's test. Differences in the proportions of Black versus White SCCS participants who would have qualified under each guideline were evaluated using Pearson's chi-square test.

Statistical analysis was performed using STATA, version 17.0 (StataCorp, College Station, TX). All *P* values were two-sided and considered statistically significant at *P* < .05.

## RESULTS

In the SCCS, 49,703 individuals met study inclusion criteria, of whom 33,585 (67.6%) were Black and 16,118 (32.4%) were White (Data Supplement, Fig S1). Baseline characteristics are shown in Table 1. During follow-up, 1,336 (4.0%) Black and 804 (5.0%) White individuals were diagnosed with primary lung cancer. As shown in Table 2, a higher proportion of Black versus White individuals with lung cancer were male, had less than a high school education, and had no comorbidities. Median age at lung cancer diagnosis was lower in Black versus White participants (61.0 years *v* 65.0 years).

**TABLE 1.** Characteristics of All Individuals With a Smoking History in the SCCS and BWHS

Characteristic	SCCS White Men and Women (n = 16,118)	SCCS Black Men and Women (n = 33,585)	BWHS Black Women (n = 22,126)
Characteristics at the time of last follow-up			
Sex, No. (%)			
Female	9,201 (57.1)	16,459 (49.0)	22,126 (100.0)
Male	6,917 (42.9)	17,126 (51.0)	
Age, years, No. (%)			
40-49	600 (3.7)	1,110 (3.3)	984 (4.5)
50-59	5,994 (37.2)	14,498 (43.2)	5,070 (22.9)
60-69	5,533 (34.3)	12,733 (37.9)	8,102 (36.6)
70-80	3,414 (21.2)	4,412 (13.1)	5,264 (23.8)
>80	577 (3.6)	832 (2.5)	2,706 (12.2)
Smoking status, No. (%)			
Formerly smoked	7,771 (48.2)	13,321 (39.7)	16,286 (73.6)
Currently smoke	8,347 (51.8)	20,264 (60.3)	5,840 (26.4)
Cigarettes per day,^a^ median (IQR)	20.0 (10.0-30.0)	10.0 (5.0-20.0)	10.0 (2.5-20.0)
Pack-years of smoking, median (IQR)	32.3 (14.8-51.0)	17.5 (7.9-31.0)	10.0 (3.38-20.0)
Smoking duration, median (IQR)	37.0 (24.0-45.0)	37.0 (27.0-44.0)	20.0 (11.0-31.0)
Years since quitting smoking, median (IQR)	22.0 (10.0-36.0)	16.0 (8.0-30.0)	27.5 (16.0-34.0)
Family history of lung cancer, No. (%)			
No	12,917 (80.1)	29,431 (87.6)	20,133 (91.0)
Yes	3,201 (19.9)	4,154 (12.4)	1,993 (9.0)
Characteristics at baseline			
BMI, median (IQR)	28.1 (24.2-33.0)	27.9 (24.0-33.0)	27.3 (23.9-31.7)
Highest level of education, No. (%)			
Less than high school	4,368 (27.1)	11,580 (34.5)	865 (3.9)
High school graduate	5,330 (33.1)	11,633 (34.6)	4,194 (19.0)
More than high school	6,413 (39.8)	10,356 (30.8)	16,904 (76.4)
Unknown	7 (<1)	16 (<1)	163 (<1)
Household income, USD, No. (%)			
<$15,000	8,387 (52.0)	21,419 (63.8)	1,262 (5.7)
≥$15,000 and <$25,000	3,016 (18.7)	7,092 (21.1)	1,604 (7.3)
≥$25,000 and <$50,000	2,412 (15.0)	3,521 (10.5)	5,338 (24.1)
≥$50,000 and <$100,000	1,561 (9.7)	1,019 (3.0)	5,501 (24.8)
≥$100,000	549 (3.4)	186 (0.6)	2,055 (9.3)
Unknown	193 (1.2)	348 (1.0)	6,366 (28.8)
Comorbidity index,^b^ No. (%)			
0	2,358 (14.6)	6,932 (20.6)	19,846 (89.7)
1	3,538 (22.0)	8,735 (26.0)	1,321 (6.0)
2	3,770 (23.4)	7,886 (23.5)	818 (3.7)
3+	6,055 (37.6)	9,474 (28.2)	141 (0.6)
Unknown	397 (2.5)	558 (1.7)	
Self-reported chronic obstructive pulmonary disease, No. (%)			Unknown
No	13,304 (82.5)	30,866 (91.9)	
Yes	2,784 (17.3)	2672 (8.0)	
Unknown	30 (0.2)	47 (0.1)	

Abbreviations: BWHS, Black Women's Health Study; SCCS, Southern Community Cohort Study.

^a^ Data on cigarettes per day were obtained from the baseline questionnaire in the SCCS and BWHS. All other smoking variables were updated in accordance with follow-up data and reflect the smoking characteristics of participants at the time of lung cancer diagnosis or last follow-up.

^b^ Comorbidity index is based on the Charlson index, with modifications to account for information available on the SCCS and BWHS baseline questionnaires.

**TABLE 2.** Characteristics of Individuals Diagnosed With Lung Cancer in the SCCS, Stratified by Race

Characteristic	White Individuals (n= 804)	Black Individuals (n = 1,336)	*P*
Sex, No. (%)			<.001
Female	461 (57.3)	570 (42.7)	
Male	343 (42.7)	766 (57.3)	
Age at diagnosis, years, median (IQR)	65.0 (58.0-71.0)	61.0 (56.0-69.0)	<.001
Smoking status at diagnosis, No. (%)			<.001
Formerly smoked	252 (31.3)	316 (23.7)	
Currently smoked	552 (68.7)	1,020 (76.3)	
Pack-year smoking history, median (IQR)	49.0 (32.1-69.9)	25.3 (16.0-42.9)	<.001
Cigarettes per day at baseline,^a^ median (IQR)	20.0 (17.0-30.0)	12.0 (9.0-20.0)	<.001
Smoking duration, median (IQR)	45.0 (38.0-51.0)	43.0 (36.0-49.0)	<.001
Years since quitting smoking, median (IQR)	11.0 (5.5-20.0)	9.0 (4.8-19.0)	.22
Highest level of education at baseline, No. (%)			<.001
Less than high school	278 (34.6)	584 (43.7)	
High school	272 (33.8)	436 (32.6)	
More than high school	252 (31.3)	314 (23.5)	
Unknown	2 (0.2)	2 (0.1)	
Household income at baseline, USD, No. (%)			<.001
<$15,000	510 (63.4)	916 (68.6)	
≥$15,000 and <$25,000	148 (18.4)	264 (19.8)	
≥$25,000 and <$50,000	81 (10.1)	106 (7.9)	
≥$50,000 and <$100,000	41 (5.1)	28 (2.1)	
≥$100,000	12 (1.5)	7 (0.5)	
Unknown	12 (1.5)	15 (1.1)	
Comorbidity index at baseline,^b^ No. (%)			<.001
0	75 (9.3)	264 (19.8)	
1	146 (18.2)	329 (24.6)	
2	189 (23.5)	310 (23.2)	
3+	375 (46.6)	411 (30.8)	
Unknown	19 (2.4)	22 (1.6)	
BMI at baseline, median (IQR)	26.8 (23.1-31.0)	25.1 (22.0-29.1)	<.001
Self-reported chronic obstructive pulmonary disease at baseline, No. (%)			<.001
No	593 (73.8)	1,182 (88.5)	
Yes	210 (26.1)	152 (11.4)	
Unknown	1 (0.1)	2 (0.1)	
Family history of lung cancer, No. (%)			<.001
No	608 (75.6)	1,162 (87.0)	
Yes	196 (24.4)	174 (13.0)	

Abbreviation: SCCS, Southern Community Cohort Study.

^a^ Data on cigarettes per day were obtained from the baseline questionnaire in the SCCS. All other smoking variables were updated in accordance with follow-up data and reflect the smoking characteristics of participants at the time of lung cancer diagnosis.

^b^ Comorbidity index is based on the Charlson index, with modifications to account for information available on the SCCS questionnaire.

### Smoking Characteristics of Individuals in the SCCS With Lung Cancer

**FIG 1.**
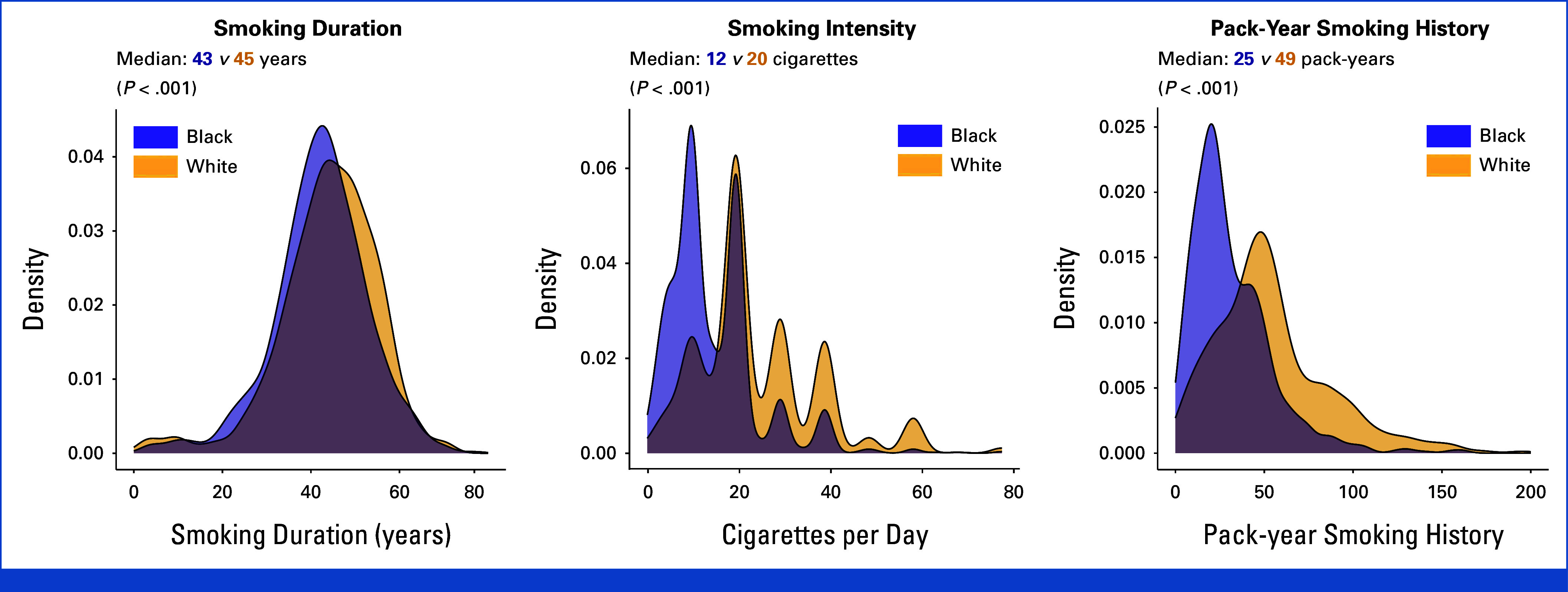
Distribution of smoking duration (years), smoking intensity (cigarettes per day), and pack-year smoking history among SCCS participants diagnosed with lung cancer. SCCS, Southern Community Cohort Study.

Black individuals with lung cancer smoked significantly fewer pack-years at lung cancer diagnosis compared with White individuals with lung cancer (median 25.3 *v* 49.0 pack-years; Fig 1). This difference was driven primarily by a difference in smoking intensity (median 12.0 *v* 20.0 cigarettes per day) rather than smoking duration (median 43.0 *v* 45.0 years).

### Screening Eligibility for Individuals in the SCCS With Lung Cancer Who Currently Smoked

**FIG 2.**
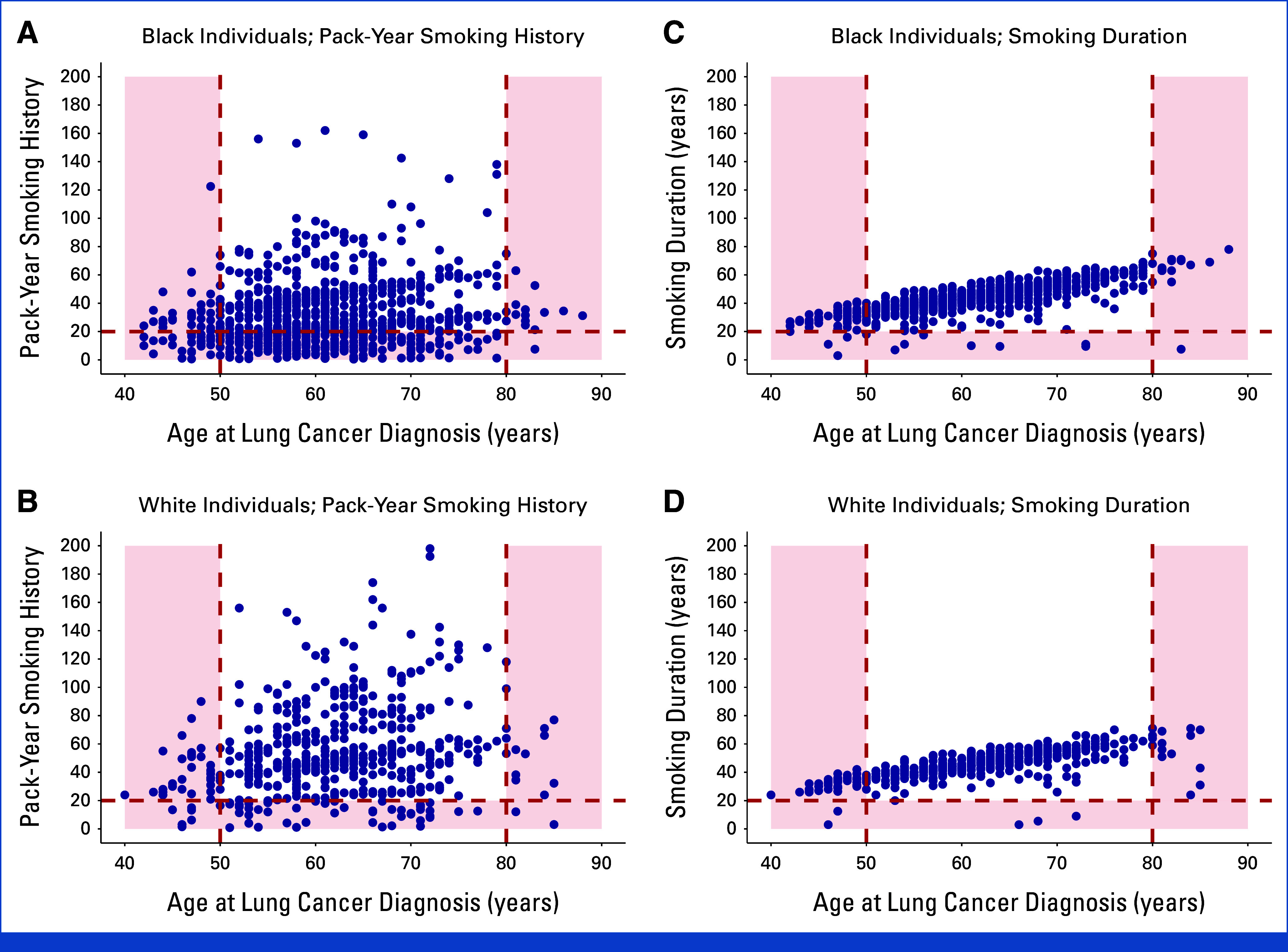
Scatterplots of pack-year smoking history versus age at lung cancer diagnosis (A and B) and smoking duration versus age at lung cancer diagnosis (C and D) among SCCS participants with lung cancer who currently smoked at the time of diagnosis, stratified by race. Each blue dot represents an individual diagnosed with lung cancer. (A and B) The red dashed lines indicate the 2021 US Preventive Services Task Force lung cancer screening eligibility criteria (ie, age 50-80 years, ≥20 pack-year smoking history). (C and D) The red dashed lines indicate the proposed lung cancer screening eligibility criteria (ie, age 50-80 years, ≥20-year smoking duration). Blue dots located in the red-shaded regions of the graph represent individuals with lung cancer who would have been ineligible for screening under each guideline. SCCS, Southern Community Cohort Study.

Figures 2A and 2B show scatterplots of pack-year smoking history versus age at lung cancer diagnosis for SCCS participants with lung cancer who currently smoked at diagnosis. Only 61.8% of Black participants would have been eligible for screening under the 2021 USPTF guideline versus 82.4% of White participants (*P* < .001).

Figures 2C and 2D show smoking duration versus age at lung cancer diagnosis for SCCS participants with lung cancer who currently smoked at diagnosis. Use of a 20-year smoking duration cutoff instead of a 20-pack-year cutoff increased the proportions of both Black and White participants who would have qualified for screening to 92.0% (*P* < .001) and 90.6% (*P* < .001), respectively, thus eliminating differences in screening eligibility between the two groups (*P* = .35).

### Screening Eligibility for Individuals in the SCCS With Lung Cancer Who Formerly Smoked

**FIG 3.**
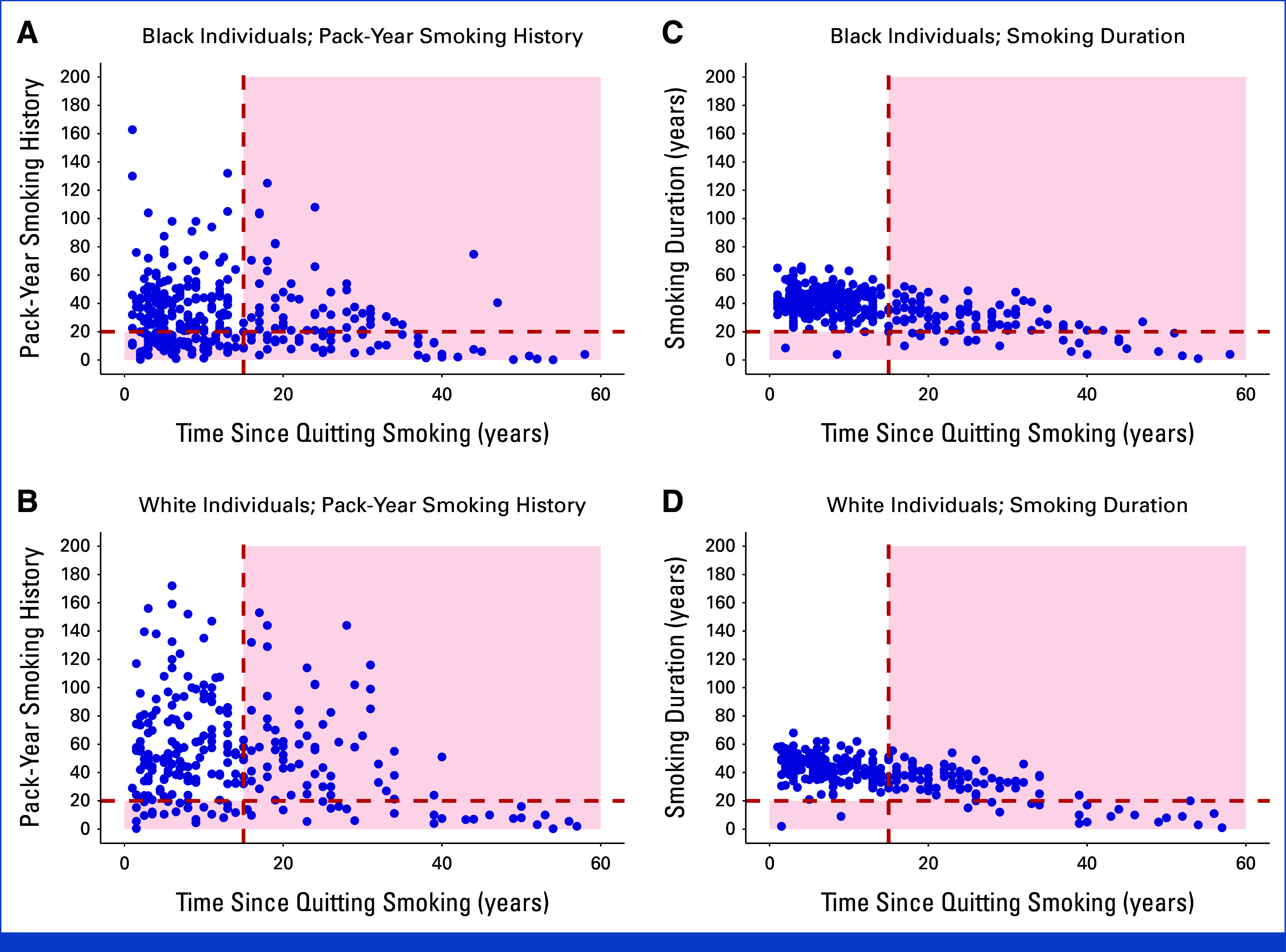
Scatterplots of pack-year smoking history versus years since quitting smoking (A and B) and smoking duration versus years since quitting smoking (C and D) among SCCS participants with lung cancer who formerly smoked at the time of diagnosis, stratified by race. Each blue dot represents an individual diagnosed with lung cancer. (A and B) The red dashed lines indicate the 2021 US Preventive Services Task Force lung cancer screening eligibility criteria (ie, quit smoking ≤15 years ago, ≥20 pack-year smoking history). (C and D) The red dashed lines indicate the proposed lung cancer screening eligibility criteria (ie, quit smoking ≤15 years ago, ≥20-year smoking duration). Blue dots located in the red-shaded regions of the graph represent individuals with lung cancer who would have been ineligible for screening under each guideline. SCCS, Southern Community Cohort Study.

Figures 3A and 3B show scatterplots of pack-year smoking history versus years since quitting smoking for SCCS participants with lung cancer who formerly smoked at diagnosis. Black participants would have been less likely to be eligible for screening under the 2021 USPSTF guideline than White participants (44.3% *v* 55.6%, respectively, *P* = .008).

Figures 3C and 3D show smoking duration versus years since quitting smoking for SCCS participants with lung cancer who formerly smoked at diagnosis. Use of a 20-year smoking duration cutoff instead of a 20-pack-year cutoff increased the proportions of both Black and White participants who would have qualified for screening to 63.9% (*P* < .001) and 63.1% (*P* < .001) respectively, thus eliminating differences in screening eligibility between the two groups (*P* = .84).

### Sensitivity of the 2021 USPSTF Guideline Versus Proposed Guideline in the SCCS

The Data Supplement (Table S2) shows the proportions of SCCS patients with lung cancer in the overall cohort who would have met each guideline. Compared with the 2021 USPSTF guideline, the proportion that would have qualified under the proposed guideline increased from 57.6% to 85.3% (*P* < .001) for Black patients and 74.0% to 82.0% (*P* < .001) for White patients, resulting in elimination of the previous disparity in screening eligibility. Of note, nearly 100% of Black and White individuals age 50-59 years diagnosed with lung cancer would have qualified for screening under the new proposed guideline (Data Supplement, Table S3). Sensitivity of the proposed guideline using different smoking duration thresholds is shown in the Data Supplement (Table S4).

### Screening Eligibility of Individuals, With or Without Lung Cancer, in the SCCS

The proportion of all SCCS participants (with or without lung cancer) who would have qualified for screening increased when the proposed guideline was used rather than the 2021 USPSTF guideline, increasing from 37.3% to 71.3% (*P* < .001) for Black SCCS participants and from 51.5% to 62.5% (*P* < .001) for White SCCS participants (Data Supplement, Table S5).

### Smoking Characteristics of Individuals Without Lung Cancer in the SCCS

The distributions of smoking patterns among SCCS participants without lung cancer who currently and formerly smoked at last follow-up are shown in the Data Supplement (Figs S2 and S3, respectively).

### Specificity of the 2021 USPSTF Guideline Versus Proposed Guideline in the SCCS

In the SCCS, specificity of the proposed guideline, as compared with the 2021 USPSTF guideline, decreased from 63.6% to 29.3% (*P <* .001) among Black individuals and from 49.7% to 38.5% (*P <* .001) among White individuals (Data Supplement, Table S2). Specificity of the proposed guideline using different smoking duration thresholds is shown in the Data Supplement (Table S4).

### Characteristics of Individuals in the SCCS Eligible for Screening

The Data Supplement (Table S6) shows characteristics of SCCS participants, with or without lung cancer, according to whether they met each of the guidelines. Compared with individuals who qualified for screening under the 2021 USPSTF guideline, individuals who qualified under the proposed guideline were more likely to be female, Black, age <60 years, and with fewer comorbidities.

### Validation Among Black Women in the BWHS

A total of 22,126 Black women in the BWHS met study inclusion criteria, among whom 486 (2.2%) were diagnosed with lung cancer during follow-up (Data Supplement, Fig S4). Baseline characteristics are shown in Table 1 for all BWHS participants and in the Data Supplement (Table S7) for patients with lung cancer. Of note, the proportion of individuals who currently smoke in the BWHS is markedly lower than that in the SCCS.

**FIG 4.**
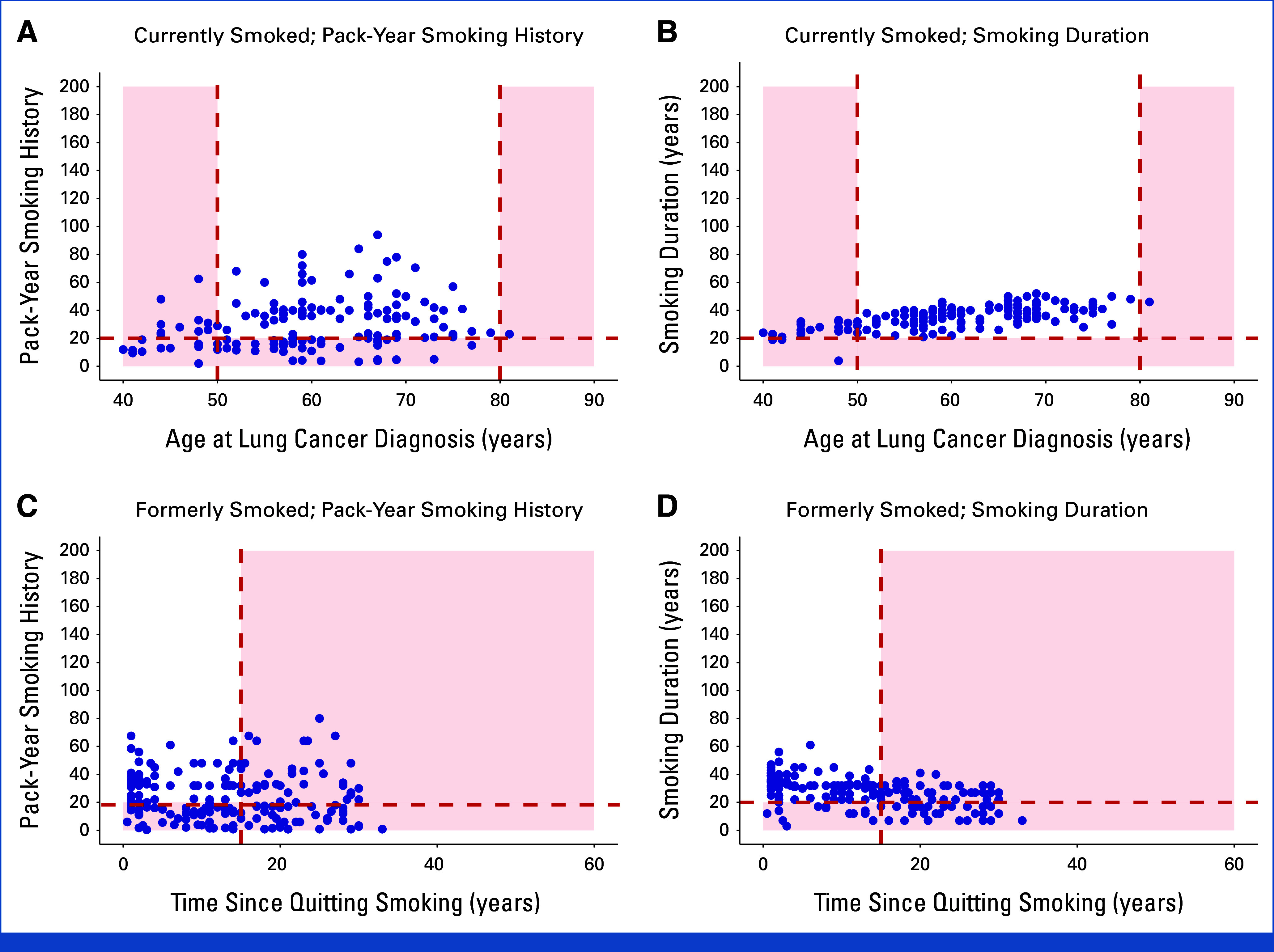
Smoking patterns among Black women with lung cancer in the BWHS. Scatterplots of (A) pack-year smoking history versus age at lung cancer diagnosis among BWHS participants with lung cancer who currently smoked at the time of diagnosis, (B) smoking duration versus age at lung cancer diagnosis among BWHS participants with lung cancer who currently smoked at the time of diagnosis, (C) pack-year smoking history versus years since quitting smoking among BWHS participants with lung cancer who formerly smoked at the time of diagnosis, and (D) smoking duration versus years since quitting smoking among BWHS participants with lung cancer who formerly smoked at the time of diagnosis. Each blue dot represents an individual diagnosed with lung cancer. Blue dots located in the red-shaded regions of the graph represent individuals with lung cancer who would have been ineligible for screening under each guideline. BWHS, Black Women's Health Study.

Figure 4A shows a scatterplot of pack-year smoking history versus age at lung cancer diagnosis for BWHS participants with lung cancer who currently smoked at diagnosis (n = 242); only 54.6% would have been eligible for screening under the 2021 USPSTF guideline. Figure 4B shows smoking duration versus age at lung cancer diagnosis for BWHS participants with lung cancer who currently smoked at diagnosis. Use of a 20-year smoking duration cutoff increased the proportion that would have qualified for screening to 82.6% (*P* < .001).

Figure 4C shows pack-year smoking history versus years since quitting smoking for BWHS participants with lung cancer who formerly smoked at diagnosis (n = 244); 30.7% would have been eligible for screening under the 2021 USPSTF guideline. Figure 4D shows smoking duration versus years since quitting smoking for BWHS participants with lung cancer who formerly smoked at diagnosis. Use of a 20-year smoking duration cutoff increased the proportion that would have qualified for screening to 45.1% (*P* < .001).

As shown in the Data Supplement (Table S2), changing to the proposed guideline increased the proportion of BWHS participants with lung cancer who would have qualified from 42.5% to 63.8% (*P* < .001). Sensitivity of the proposed guideline using different smoking duration thresholds is shown in the Data Supplement (Table S4).

While 13.9% of all BWHS participants (with or without lung cancer) who currently or formerly smoked would have qualified for screening under the 2021 USPSTF guideline, changing the guideline would increase the proportion to 29.1% (*P* < .001).

Finally, specificity decreased from 86.8% for the 2021 USPSTF guideline to 71.7% for the proposed guideline (*P <* .001) among BWHS participants (Data Supplement, Table S2). Specificity of the proposed guideline using different smoking duration thresholds is shown in the Data Supplement (Table S4).

## DISCUSSION

In the present analysis of data from two large prospective cohort studies, we found that 58% of Black patients with lung cancer in the SCCS, 74% of White patients with lung cancer in the SCCS, and 43% of Black women with lung cancer in the BWHS would have been eligible for screening under the 2021 USPSTF lung cancer screening guideline. Revising that guideline to include a 20-year smoking duration cutoff instead of a 20-pack-year cutoff increased the proportion of individuals with lung cancer who would have qualified for screening to over 80% for both Black and White individuals in the SCCS (thus eliminating the racial disparity in screening eligibility) and to 64% for Black women in the BWHS.

In the United States, pack-year smoking history is routinely used to determine lung cancer screening eligibility. This criterion emerged from findings of the NLST—the landmark randomized trial which showed that LDCT screening resulted in a 20% reduction in lung cancer mortality compared with chest X-ray screening.^2^ Since then, the USPSTF,^13^ Centers for Medicare and Medicaid Services,^32^ National Comprehensive Cancer Network,^33^ and major medical societies^34^ have all recommended lung cancer screening for individuals who have smoked at least 20 pack-years.

Our proposed guideline—which uses a 20-year smoking duration cutoff instead of a 20-pack-year smoking history cutoff—has several important advantages. First, use of the new cutoff increased the proportion of patients with lung cancer who would have qualified for screening to over 80% for both Black and White SCCS participants and 64% for Black women in the BWHS. Second, the proposed guideline eliminated the racial disparity in screening eligibility between Black versus White individuals. To date, the key effort to reduce racial disparities in lung cancer screening eligibility was the revision of the 2013 USPSTF guideline to lower the age limit and reduce the number of smoking pack-years required.^13,35^ However, our findings, in addition to the findings of several previous studies,^4-11^ demonstrate that these previous changes were unsuccessful in eliminating racial disparities in screening eligibility. Importantly, revising the guideline to include a 20-year smoking duration cutoff instead of a 20-pack-year cutoff completely eliminated the racial disparity between Black and White individuals in screening eligibility. These data suggest that revising the USPSTF criteria to include a 20-year smoking duration cutoff instead of a 20-pack-year cutoff would be an important step forward to eliminate racial disparities in lung cancer screening eligibility and would contribute to the advancement of ongoing efforts by the USPSTF to address systemic racism in preventive service recommendations.^67^

Third, the proposed guideline is simple and easy to implement. The use of more complex lung cancer risk prediction models has been explored as a possible strategy to improve the selection of individuals for screening and to reduce racial disparities in screening eligibility.^8,36-40^ However, the USPSTF has expressed concerns over the use of risk prediction models as they may impose a barrier to wider implementation and uptake of lung cancer screening,^3^ especially given that uptake of lung cancer screening in the United States is already extremely low (<5% of eligible individuals underwent screening in 2022).^41^ A notable strength of the proposed guideline is that it is simple—even simpler than the current 2021 USPSTF guideline—as calculating the number of years someone has smoked is easier to do and more precise than calculating pack-year smoking history.^42-44^ In addition, while most individuals can report the exact year they started and/or stopped smoking—thereby allowing for very precise calculations of smoking duration—most individuals do not smoke the same number of cigarettes per day for the entirety of their smoking history.^45^ Fourth, using a 20-year smoking duration cutoff instead of a 20-pack-year cutoff predominately increased screening eligibility for younger individuals, among whom the benefit of early detection and access to curative-intent therapy is likely to be greatest.^46^ Notably, nearly 100% of Black and White individuals in the SCCS age 50-59 years with lung cancer would have qualified for screening under the proposed guideline.

When compared with the 2021 USPSTF criteria, specificity of the proposed guideline was lower, especially among Black SCCS participants. At first glance, the lower specificity of the proposed guideline might suggest that the proposed guideline will expand screening to many low-risk individuals who will never develop lung cancer. However, there are several important factors to consider in interpreting these changes in specificity. First, the proposed guideline predominately expanded screening eligibility to younger individuals age 50-59 years who smoked for long durations but less intensely; individuals who have smoked long durations but less intensely (ie, 1-10 cigarettes per day) have been shown to have a notably increased risk of lung cancer death—nearly 12-fold higher compared with individuals who have never smoked.^45^ Although the majority of these individuals did not have lung cancer at the time of last follow-up (and thus were classified as false positives, resulting in lower specificity), many of these individuals have a high risk of developing lung cancer in the future.

Second, it is important to consider the differences in specificity between the SCCS and BWHS. While in the SCCS, specificity dropped from 63.6% to 29.3% among Black individuals, in the BWHS, there was only a small drop, from 86.8% to 71.7%. This difference is attributed to differences in the smoking patterns of participants in the two cohorts. The SCCS comprises a very high-risk population: 71.3% of participants without lung cancer either actively smoke or recently (within the past 15 years) quit smoking and have smoked for ≥20 years. As a result, many SCCS participants without lung cancer qualified for screening under the proposed guideline and were classified as false positives, resulting in low specificity of the proposed guideline in the SCCS. By contrast, in the BWHS, there is a greater proportion of individuals with shorter, remote smoking histories. The incidence of lung cancer in these individuals has been shown to be low.^47^ As a result, many BWHS participants without lung cancer did not qualify for screening under the proposed guideline and were classified as true negatives, resulting in high specificity of the proposed guideline in the BWHS. In the general US population, smoking patterns are more similar to what is observed in the BWHS, rather than the SCCS.^48,49^ It is likely that specificity of the proposed guideline in the general US population would be much higher than that in the SCCS and would be more similar to the specificity observed in the BWHS.

We explored the impact of increasing the smoking duration threshold in the proposed guideline. While higher duration thresholds increased specificity in the SCCS and BWHS, those thresholds excluded many younger (age 50-59 years) individuals who recently quit smoking (and thus had not reached ≥30 years of smoking, for example), many of whom likely have a high risk of developing lung cancer in the future. Notably, use of higher duration thresholds led to sharp drops in sensitivity of the proposed guideline in the BWHS: sensitivity of the proposed guideline using a 30- and 40-year duration threshold in the BWHS was only 50.6% and 17.7%, respectively.

Given the increases in eligibility observed under the proposed guideline, particularly among Black individuals, it is worth considering how these changes may affect the potential harms of lung cancer screening on the population. Harms of lung cancer screening have been shown to be very low overall and include false-positive findings leading to unnecessary tests, invasive procedures, and treatment,^46^^,^^50-55^ lung cancer overdiagnosis,^46,50^ and radiation-related lung cancer deaths.^46^ Under the proposed guideline, a higher proportion of Black individuals will likely become eligible than previously and are therefore at risk for these harms. They will not, however, be at greater risk of harm than White individuals. Furthermore, the very low risk of harm from lung cancer screening must be considered, as always, in the context of the life-saving potential of early lung cancer detection.

Finally, it is important to recognize the structural factors, apart from eligibility criteria, that prevent high-risk Black individuals from being screened for lung cancer and that contribute to racial disparities in lung cancer screening use. Notable barriers to lung cancer screening include a lack of awareness of screening and difficulties in accessing lung cancer screening (eg, the inability to take time off of work to get screening).^56-59^ These barriers have been shown to disproportionately affect individuals from racial minority groups.^60,61^ In addition, challenges in identifying patients who are eligible for screening because of missing or inaccurate pack-year smoking history documented in the electronic medical record have been shown to be key barriers to the uptake of lung cancer screening.^42-44,62^ In addition to efforts to make screening eligibility criteria more equitable, efforts to address these structural factors are needed. We believe that the use of a smoking duration cutoff, rather than a pack-year cutoff, may help in this regard by simplifying the guideline and making it easier to identify eligible individuals.

There are limitations to this study. First, exposure misclassification is a possibility because smoking information is self-reported. However, both the SCCS and BWHS have published multiple papers associating cigarette smoking, including duration and/or pack-years, with the risk of various health outcomes, with findings as expected from the literature.^63-66^ Second, individuals in the SCCS and BWHS may not be representative of all individuals at high risk for lung cancer in the United States; the sensitivity and specificity of the proposed guideline may differ in other populations. A notable strength, however, of using both cohorts for this analysis is that they represent different regions of the United States, different socioeconomic backgrounds, and markedly different smoking patterns, with more individuals who currently smoke in the SCCS than in the BWHS.

In conclusion, in this analysis of two large cohort studies, we found that using a 20-pack-year smoking history cutoff as a selection criterion for lung cancer screening excluded many individuals diagnosed with lung cancer and led to a marked racial disparity in screening eligibility between Black versus White individuals. Revising the USPSTF guideline to include a 20-year smoking duration cutoff (instead of a 20-pack-year smoking history cutoff) increased the proportion of patients with lung cancer who would qualify for screening and eliminated the racial disparity in screening eligibility. These findings challenge the use of pack-year smoking history in determining lung cancer screening eligibility and support the use of smoking duration cutoffs instead as a simple yet effective change to increase the sensitivity of the guideline and improve equity in opportunities for screening.

## References

[1] de Koning HJ, van der Aalst CM, de Jong PA, et al: Reduced lung-cancer mortality with volume CT screening in a randomized trial. N Engl J Med 382:503-513, 2020 31995683 10.1056/NEJMoa1911793

[2] National Lung Screening Trial Research Team, Aberle DR, Adams AM, et al: Reduced lung-cancer mortality with low-dose computed tomographic screening. N Engl J Med 365:395-409, 2011 21714641 10.1056/NEJMoa1102873 PMC4356534

[3] US Preventive Services Task Force, Krist AH, Davidson KW, et al: Screening for lung cancer: US Preventive Services Task Force recommendation statement. JAMA 325:962-970, 2021 33687470 10.1001/jama.2021.1117

[4] Potter AL, Yang C-FJ, Woolpert KM, et al: Evaluating eligibility of US Black women under USPSTF lung cancer screening guidelines. JAMA Oncol 8:163-164, 2022 34817564 10.1001/jamaoncol.2021.5790 PMC8777560

[5] Liu A, Siddiqi N, Tapan U, et al: Black race remains associated with lower eligibility for screening using 2021 US Preventive Services Task Force recommendations among lung cancer patients at an urban safety net hospital. J Racial Ethn Health Disparities 10:2836-2843, 2022 36441493 10.1007/s40615-022-01460-x PMC12812330

[6] Reese TJ, Schlechter CR, Potter LN, et al: Evaluation of revised US Preventive Services Task Force lung cancer screening guideline among women and racial/ethnic minority populations. JAMA Netw Open 4:e2033769, 2021 33433600 10.1001/jamanetworkopen.2020.33769 PMC7804914

[7] Pinheiro LC, Groner L, Soroka O, et al: Analysis of eligibility for lung cancer screening by race after 2021 changes to US preventive Services Task Force screening guidelines. JAMA Netw Open 5:e2229741, 2022 36053535 10.1001/jamanetworkopen.2022.29741 PMC9440399

[8] Williams RM, Kareff SA, Sackstein P, et al: Race & sex disparities related to low-dose computed tomography lung cancer screening eligibility criteria: A lung cancer cases review. Lung Cancer 169:55-60, 2022 35644087 10.1016/j.lungcan.2022.05.008 PMC9248363

[9] Maki KG, Talluri R, Toumazis I, et al: Impact of U.S. Preventive Services Task Force lung cancer screening update on drivers of disparities in screening eligibility. Cancer Med 12:4647-4654, 2023 35871312 10.1002/cam4.5066 PMC9972155

[10] Smeltzer MP, Liao W, Faris NR, et al: Potential impact of criteria modifications on race and sex disparities in eligibility for lung cancer screening. J Thorac Oncol 18:158-168, 2023 36208717 10.1016/j.jtho.2022.09.220

[11] Potter AL, Senthil P, Srinivasan D, et al: Persistent race- and sex-based disparities in lung cancer screening eligibility. J Thorac Cardiovasc Surg 10.1016/j.jtcvs.2023.10.025 [epub ahead of print on October 18, 2023] 10.1016/j.jtcvs.2023.10.025 PMC11698625 37863179

[12] Pleasants RA, Rivera MP, Tilley SL, et al: Both duration and pack-years of tobacco smoking should be used for clinical practice and research. Ann Am Thorac Soc 17:804-806, 2020 32348693 10.1513/AnnalsATS.202002-133VP PMC7405110

[13] US Preventive Services Task Force: Lung Cancer: Screening. https://www.uspreventiveservicestaskforce.org/uspstf/recommendation/lung-cancer-screening

[14] Reference deleted

[15] Reference deleted

[16] Reference deleted

[17] Reference deleted

[18] Reference deleted

[19] Peto J: That the effects of smoking should be measured in pack-years: Misconceptions 4. Br J Cancer 107:406-407, 2012 22828655 10.1038/bjc.2012.97 PMC3405232

[20] Vlaanderen J, Portengen L, Schüz J, et al: Effect modification of the association of cumulative exposure and cancer risk by intensity of exposure and time since exposure cessation: A flexible method applied to cigarette smoking and lung cancer in the SYNERGY study. Am J Epidemiol 179:290-298, 2014 24355332 10.1093/aje/kwt273 PMC3895097

[21] Remen T, Pintos J, Abrahamowicz M, et al: Risk of lung cancer in relation to various metrics of smoking history: A case-control study in montreal. BMC Cancer 18:1275, 2018 30567516 10.1186/s12885-018-5144-5 PMC6299933

[22] Flanders WD, Lally CA, Zhu BP, et al: Lung cancer mortality in relation to age, duration of smoking, and daily cigarette consumption: Results from cancer prevention study II. Cancer Res 63:6556-6562, 2003 14559851

[23] Lubin JH, Caporaso NE: Cigarette smoking and lung cancer: Modeling total exposure and intensity. Cancer Epidemiol Biomarkers Prev 15:517-523, 2006 16537710 10.1158/1055-9965.EPI-05-0863

[24] Doll R, Peto R: Cigarette smoking and bronchial carcinoma: Dose and time relationships among regular smokers and lifelong non-smokers. J Epidemiol Community Health 32:303-313, 1978 744822 10.1136/jech.32.4.303 PMC1060963

[25] Aldrich MC, Mercaldo SF, Sandler KL, et al: Evaluation of USPSTF lung cancer screening guidelines among African American adult smokers. JAMA Oncol 5:1318-1324, 2019 31246249 10.1001/jamaoncol.2019.1402 PMC6604090

[26] Trinidad DR, Pérez-Stable EJ, White MM, et al: A nationwide analysis of US racial/ethnic disparities in smoking behaviors, smoking cessation, and cessation-related factors. Am J Public Health 101:699-706, 2011 21330593 10.2105/AJPH.2010.191668 PMC3052356

[27] Trinidad DR, Pérez-Stable EJ, Emery SL, et al: Intermittent and light daily smoking across racial/ethnic groups in the United States. Nicotine Tob Res 11:203-210, 2009 19246433 10.1093/ntr/ntn018 PMC2658897

[28] Center for Disease Control: United States Cancer Statistics: Data Visualizations. https://gis.cdc.gov/Cancer/USCS/DataViz.html

[29] Potter AL, Rosenstein AL, Kiang MV, et al: Association of computed tomography screening with lung cancer stage shift and survival in the United States: Quasi-experimental study. BMJ 376:e069008, 2022 35354556 10.1136/bmj-2021-069008 PMC8965744

[30] Zullig LL, Carpenter WR, Provenzale DT, et al: The association of race with timeliness of care and survival among Veterans Affairs health care system patients with late-stage non-small cell lung cancer. Cancer Manag Res 5:157-163, 2013 23900515 10.2147/CMAR.S46688 PMC3726302

[31] SEER: Site Recode ICD-O-3/WHO 2008 Definition. https://seer.cancer.gov/siterecode/icdo3_dwhoheme/index.html

[32] CMS: Screening for Lung Cancer with Low Dose Computed Tomography (LDCT) CAG-00439R. 2022. https://www.cms.gov/medicare-coverage-database/view/ncacal-decision-memo.aspx?proposed=N&ncaid=304

[33] NCCN (National Comprehensive Cancer Network): NCCN Clinical Practice Guidelines in Oncology (NCCN Guidelines): Lung Cancer Screening, Version 1.2023—October 26. 2022. https://www.nccn.org/professionals/physician_gls/pdf/lung_screening.pdf

[34] Mazzone PJ, Silvestri GA, Souter LH, et al: Screening for lung cancer: CHEST guideline and expert panel report. Chest 160:e427-e494, 2021 34270968 10.1016/j.chest.2021.06.063 PMC8727886

[35] US Preventive Services Task Force: Lung Cancer: Screening. 2013. https://uspreventiveservicestaskforce.org/uspstf/recommendation/lung-cancer-screening-december-2013

[36] Pasquinelli MM, Tammemägi MC, Kovitz KL, et al: Addressing sex disparities in lung cancer screening eligibility: USPSTF vs PLCOm2012 criteria. Chest 161:248-256, 2022 34252436 10.1016/j.chest.2021.06.066

[37] Tammemägi MC, Ruparel M, Tremblay A, et al: USPSTF2013 versus PLCOm2012 lung cancer screening eligibility criteria (International Lung Screening Trial): Interim analysis of a prospective cohort study. Lancet Oncol 23:138-148, 2022 34902336 10.1016/S1470-2045(21)00590-8 PMC8716337

[38] Pasquinelli MM, Tammemägi MC, Kovitz KL, et al: Risk prediction model versus United States preventive Services Task Force lung cancer screening eligibility criteria: Reducing race disparities. J Thorac Oncol 15:1738-1747, 2020 32822843 10.1016/j.jtho.2020.08.006

[39] Aredo JV, Choi E, Ding VY, et al: Racial and ethnic disparities in lung cancer screening by the 2021 USPSTF guidelines versus risk-based criteria: The multiethnic cohort study. JNCI Cancer Spectr 6:pkac033, 2022 35642317 10.1093/jncics/pkac033 PMC9156850

[40] Choi E, Ding VY, Luo SJ, et al: Risk model–based lung cancer screening and racial and ethnic disparities in the US. JAMA Oncol 9:1640-1648, 2023 37883107 10.1001/jamaoncol.2023.4447 PMC10603577

[41] American Lung Association: State of Lung Cancer: Key Findings. https://www.lung.org/research/state-of-lung-cancer/key-findings

[42] Kukhareva PV, Caverly TJ, Li H, et al: Inaccuracies in electronic health records smoking data and a potential approach to address resulting underestimation in determining lung cancer screening eligibility. J Am Med Inform Assoc 29:779-788, 2022 35167675 10.1093/jamia/ocac020 PMC9006678

[43] Modin HE, Fathi JT, Gilbert CR, et al: Pack-year cigarette smoking history for determination of lung cancer screening eligibility. Comparison of the electronic medical record versus a shared decision-making conversation. Ann Am Thorac Soc 14:1320-1325, 2017 28406708 10.1513/AnnalsATS.201612-984OC

[44] Kinsinger LS, Anderson C, Kim J, et al: Implementation of lung cancer screening in the veterans health administration. JAMA Intern Med 177:399-406, 2017 28135352 10.1001/jamainternmed.2016.9022

[45] Inoue-Choi M, Liao LM, Reyes-Guzman C, et al: Association of long-term, low-intensity smoking with all-cause and cause-specific mortality in the national Institutes of health-AARP diet and health study. JAMA Intern Med 177:87-95, 2017 27918784 10.1001/jamainternmed.2016.7511 PMC5555224

[46] Meza R, Jeon J, Toumazis I, et al: Evaluation of the benefits and harms of lung cancer screening with low-dose computed tomography: Modeling study for the US preventive Services Task Force. JAMA 325:988-997, 2021 33687469 10.1001/jama.2021.1077 PMC9208912

[47] Bach PB, Kattan MW, Thornquist MD, et al: Variations in lung cancer risk among smokers. J Natl Cancer Inst 95:470-478, 2003 12644540 10.1093/jnci/95.6.470

[48] Kramarow EA: Health of former cigarette smokers aged 65 and over: United States, 2018. Natl Health Stat Rep 145:1-12, 2020 32730739

[49] Mayer M, Reyes-Guzman C, Grana R, et al: Demographic characteristics, cigarette smoking, and e-cigarette use among US adults. JAMA Netw Open 3:e2020694, 2020 33048127 10.1001/jamanetworkopen.2020.20694 PMC8094416

[50] Hoffman RM, Atallah RP, Struble RD, et al: Lung cancer screening with low-dose CT: A meta-analysis. J Gen Intern Med 35:3015-3025, 2020 32583338 10.1007/s11606-020-05951-7 PMC7573097

[51] Erkmen CP, Randhawa S, Patterson F, et al: Quantifying benefits and harms of lung cancer screening in an underserved population: Results from a prospective study. Semin Thorac Cardiovasc Surg 34:691-700, 2022 34091014 10.1053/j.semtcvs.2021.04.055 PMC8645668

[52] Kaminetzky M, Milch HS, Shmukler A, et al: Effectiveness of lung-RADS in reducing false-positive results in a diverse, underserved, urban lung cancer screening cohort. J Am Coll Radiol 16:419-426, 2019 30146484 10.1016/j.jacr.2018.07.011

[53] Erkmen CP, Dako F, Moore R, et al: Adherence to annual lung cancer screening with low-dose CT scan in a diverse population. Cancer Causes Control 32:291-298, 2021 33394208 10.1007/s10552-020-01383-0 PMC7878339

[54] Kamel MK, Kariyawasam S, Stiles B: Overestimation of screening-related complications in the National Lung Screening trial. J Thorac Cardiovasc Surg 166:336-344.e2, 2023 36503729 10.1016/j.jtcvs.2022.10.051

[55] Manyak A, Seaburg L, Bohreer K, et al: Invasive procedures associated with lung cancer screening in clinical practice. Chest 164:544-555, 2023 36781101 10.1016/j.chest.2023.02.010

[56] Draucker CB, Rawl SM, Vode E, et al: Understanding the decision to screen for lung cancer or not: A qualitative analysis. Health Expect 22:1314-1321, 2019 31560837 10.1111/hex.12975 PMC6882261

[57] Carter-Harris L, Gould MK: Multilevel barriers to the successful implementation of lung cancer screening: Why does it have to be so hard? Ann Am Thorac Soc 14:1261-1265, 2017 28541749 10.1513/AnnalsATS.201703-204PS

[58] Tseng TS, Gross T, Celestin MD, et al: Knowledge and attitudes towards low dose computed tomography lung cancer screening and smoking among African Americans-a mixed method study. Transl Cancer Res 8:S431-S442, 2019 35117119 10.21037/tcr.2019.04.18 PMC8797997

[59] Williams RM, Beck KH, Butler J III, et al: Lung cancer screening decisional needs among African American smokers of lower socioeconomic status. Ethn Health 27:565-583, 2022 32498546 10.1080/13557858.2020.1771681 PMC7718398

[60] Carter-Harris L, Slaven JE Jr, Monahan PO, et al: Understanding lung cancer screening behavior: Racial, gender, and geographic differences among Indiana long-term smokers. Prev Med Rep 10:49-54, 2018 29552458 10.1016/j.pmedr.2018.01.018 PMC5852404

[61] Barta JA, Shusted CS, Ruane B, et al: Racial differences in lung cancer screening beliefs and screening adherence. Clin Lung Cancer 22:570-578, 2021 34257020 10.1016/j.cllc.2021.06.003

[62] Zeliadt SB, Hoffman RM, Birkby G, et al: Challenges implementing lung cancer screening in federally qualified health centers. Am J Prev Med 54:568-575, 2018 29429606 10.1016/j.amepre.2018.01.001 PMC8483158

[63] Blot WJ, Cohen SS, Aldrich M, et al: Lung cancer risk among smokers of menthol cigarettes. J Natl Cancer Inst 103:810-816, 2011 21436064 10.1093/jnci/djr102 PMC3096798

[64] Aldrich MC, Munro HM, Mumma M, et al: Chronic obstructive pulmonary disease and subsequent overall and lung cancer mortality in low-income adults. PLoS One 10:e0121805, 2015 25811837 10.1371/journal.pone.0121805 PMC4374870

[65] Coogan PF, Castro-Webb N, Yu J, et al: Active and passive smoking and the incidence of asthma in the Black Women's Health Study. Am J Respir Crit Care Med 191:168-176, 2015 25387276 10.1164/rccm.201406-1108OC PMC4347433

[66] Formica MK, Palmer JR, Rosenberg L, et al: Smoking, alcohol consumption, and risk of systemic lupus erythematosus in the Black Women's Health Study. J Rheumatol 30:1222-1226, 2003 12784393

[67] Doubeni CA, Simon M, Krist AH: Addressing systemic racism through clinical preventive service recommendations from the US Preventive Services Task Force. JAMA 325:627-628, 2021 33492333 10.1001/jama.2020.26188

