# Supplementary material for: Pack-Year Smoking History: An Inadequate and Biased Measure to Determine Lung Cancer Screening Eligibility
Source: J Clin Oncol. Author manuscript; Available in PMC 2024 Jul 15. (PMC11191064; doi:10.1200/JCO.23.01780)
Supplement: Supplementary Appendix [file NIHMS2001341-supplement-Supplementary_Appendix.pdf]

Supplementary Appendix

Pack-Year Smoking History: An Inadequate and Biased Measure to Determine Lung Cancer Screening Eligibility

	Page
Methodology	2
Supplemental Figure 1	5
Supplemental Figure 2	6
Supplemental Figure 3	7
Supplemental Figure 4	8
Supplemental Table 1	9
Supplemental Table 2	11
Supplemental Table 3	12
Supplemental Table 4	13
Supplemental Table 5	14
Supplemental Table 6	15
Supplemental Table 7	17
References	18

Methods.

Data Sources:

Southern Community Cohort Study

The Southern Community Cohort Study (SCCS) is a large prospective cohort study that enrolled nearly 85,000 predominately low-income Black and white adults from 12 states in the southeastern United States (including Alabama, Arkansas, Florida, Georgia, Kentucky, Louisiana, Mississippi, North Carolina, South Carolina, Tennessee, Virginia, and West Virginia) from March 25th, 2002 to September 24th, 2009.[1] Individuals who enrolled in the SCCS completed a baseline questionnaire and up to three additional follow-up questionnaires, each with detailed information on cigarette smoking characteristics (i.e., smoking status, duration, and intensity). Incident lung cancer cases diagnosed among individuals in the SCCS were collected up to December 31st, 2021. Informed consent was obtained from each individual who enrolled into the SCCS and the Institutional Review Boards at Vanderbilt University (Nashville, TN, USA) and Meharry Medical College (Nashville, TN, USA) approved the study.

Black Women's Health Study

The Black Women's Health Study (BWHS) is the largest prospective cohort study of Black women in the United States.[2] In 1995, 58,973 self-identified Black women from predominately metropolitan regions across the U.S. enrolled in the BWHS by completing a health questionnaire with detailed information on cigarette smoking and other exposures. Information on cigarette smoking and other exposures was updated by biennial questionnaires.[2] Incident lung cancer cases diagnosed among individuals in the BWHS were identified by self-report and annual linkages with state cancer registries and the National Death Index up to December 31st, 2021. Informed consent was obtained from each individual who enrolled into the BWHS and the Institutional Review Board at Boston University (Boston, MA, USA) approved the study.

Definition of Smoking Characteristics:

The same methodology was used to define smoking characteristics (i.e., smoking status, cumulative years smoked, smoking intensity, and pack-year smoking history) in the Southern Community Cohort Study (SCCS) and Black Women's Health Study (BWHS).

Smoking Status: Smoking status (i.e., never, former, or current) was obtained from the smoking status reported on the most recent follow-up questionnaire prior to lung cancer diagnosis (for participants diagnosed with lung cancer) or the smoking status reported on the last follow-up questionnaire (for participants never diagnosed with lung cancer). If participants had missing data for smoking status reported on a follow-up questionnaire, their smoking status was assumed to be the same as their last known smoking status.

In the SCCS, study participants who had never smoked were considered to be individuals who self-reported that they smoked fewer than 100 cigarettes in their lifetime on the baseline SCCS questionnaire and did not report starting smoking on any of the follow-up questionnaires. In the BWHS, study participants who had never smoked were considered to be women who reported that they had never smoked one cigarette or more every day for at least a year and did not report starting smoking on any of the follow-up questionnaires.

Smoking Duration: Smoking duration was calculated based on the number of years smoked at study enrollment and was updated in accordance with follow-up data. If a participant quit smoking during follow-up, the total number of years smoked prior to that timepoint was added to 0.5 times the number of years elapsed between the last questionnaire where they reported actively smoking and the questionnaire where they reported they had quit smoking. Alternatively, if a participant started smoking during follow-up, the total number of years smoked (if any) prior to that timepoint was added to 0.5 times the number of years elapsed between the last questionnaire where they reported not actively smoking and the questionnaire where they reported they had begun actively smoking.

Smoking Intensity: Smoking intensity was determined based on the average number of cigarettes smoked per day reported at study enrollment. If participants had unknown or missing smoking intensity reported at study enrollment (i.e., because they did not smoke at the time of study enrollment but subsequently started smoking during follow-up), smoking intensity was obtained from the questionnaire where they first reported actively smoking.

Pack-year Smoking History: Pack-year smoking history was calculated by multiplying smoking duration by the average number of cigarette packages smoked per day (defined as smoking intensity divided by 20).

Years Since Quitting Smoking: Among participants who had quit smoking and were diagnosed with lung cancer, the number of years since quitting smoking was defined as the number of years elapsed between the age at which they quit smoking and lung cancer diagnosis. Among participants who had quit smoking and were not diagnosed with lung cancer, the number of years since quitting smoking was defined as the number of years that elapsed between the age at which they quit smoking and the date of the last known follow-up. For both participants with and without lung cancer, the age at which participants quit smoking was either the age they reported quitting (if they quit prior to enrollment into the SCCS or BWHS) or the midway point between the age they last reported smoking and the age they first reported they no longer smoked.

Supplementary Figure 1. Flow Diagram for Participants in the Southern Community Cohort Study (SCCS)

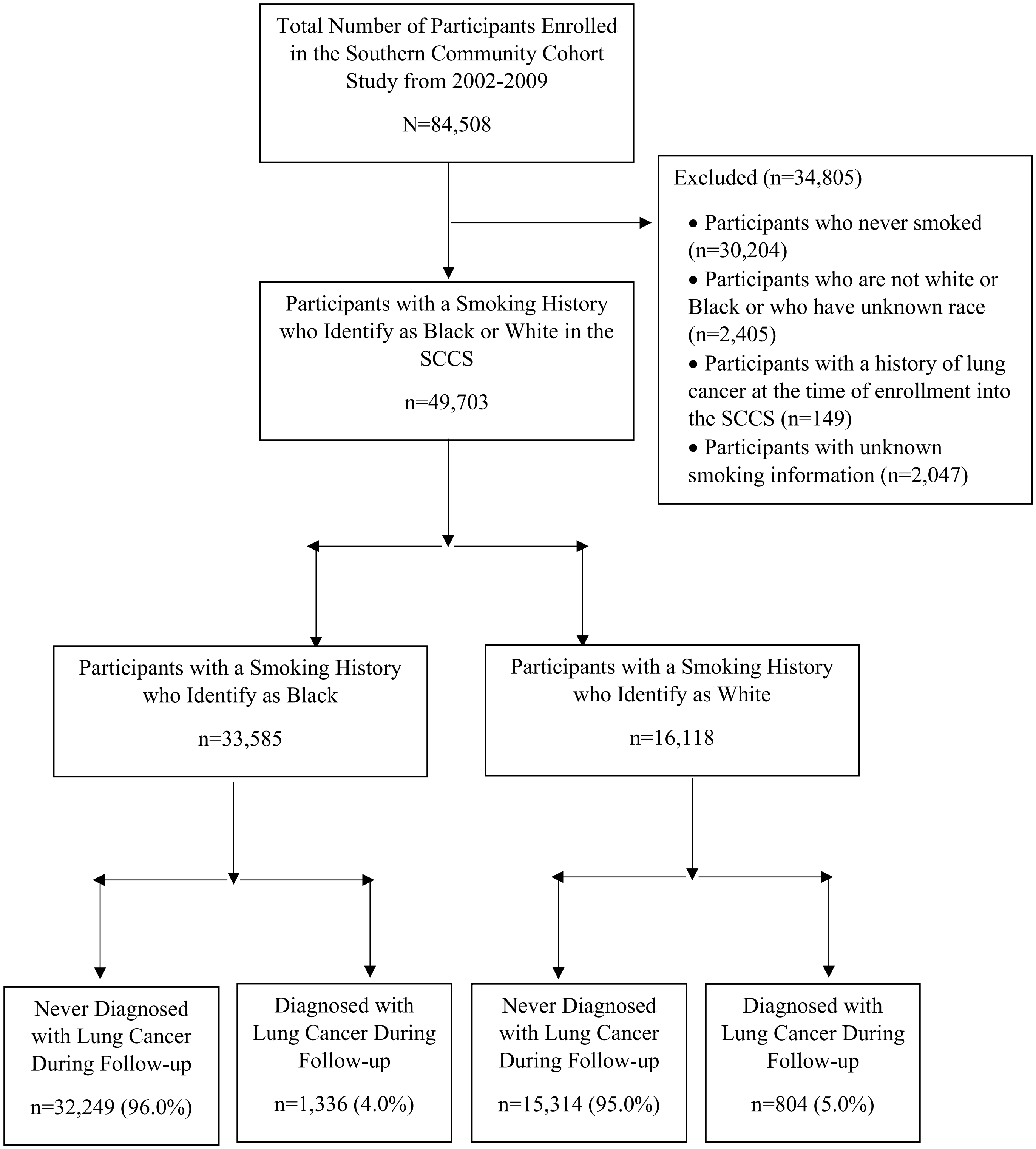

Supplementary Figure 2. Scatterplots of pack-year smoking history versus age at last follow-up (A and B) and smoking duration versus age at last follow-up (C and D) among SCCS participants without lung cancer who currently smoked at the time of last follow-up, stratified by race. Each blue dot represents one SCCS participant without lung cancer. In A and B, the red dashed lines indicate the 2021 United States Preventive Services Task Force lung cancer screening eligibility criteria (i.e., aged 50-80, $\geq$ 20 pack-year smoking history). In C and D, the red dashed lines indicate the proposed lung cancer screening eligibility criteria (i.e., aged 50-80, $\geq$ 20-year smoking duration). Blue dots located in the red-shaded regions of the graph represent SCCS participants who would have been ineligible for screening under each guideline.

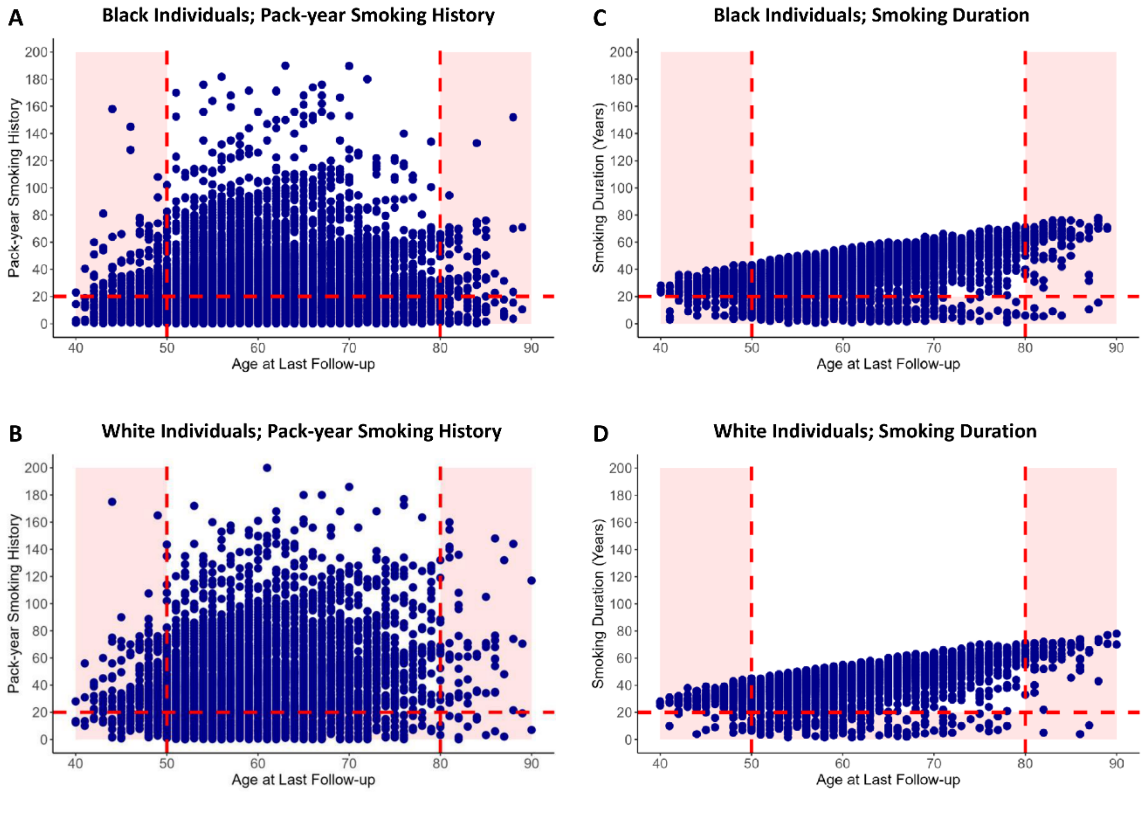

Supplementary Figure 3. Scatterplots of pack-year smoking history versus years since quitting smoking (A and B) and smoking duration versus years since quitting smoking (C and D) among SCCS participants without lung cancer who formerly smoked at the time of last follow-up, stratified by race. Each blue dot represents one SCCS participant without lung cancer. In A and B, the red dashed lines indicate the 2021 United States Preventive Services Task Force lung cancer screening eligibility criteria (i.e., quit smoking ≤ 15 years ago, ≥ 20 pack-year smoking history). In C and D, the red dashed lines indicate the proposed lung cancer screening eligibility criteria (i.e., quit smoking ≤ 15 years ago, ≥ 20-year smoking duration). Blue dots located in the red-shaded regions of the graph represent SCCS participants who would have been ineligible for screening under each guideline.

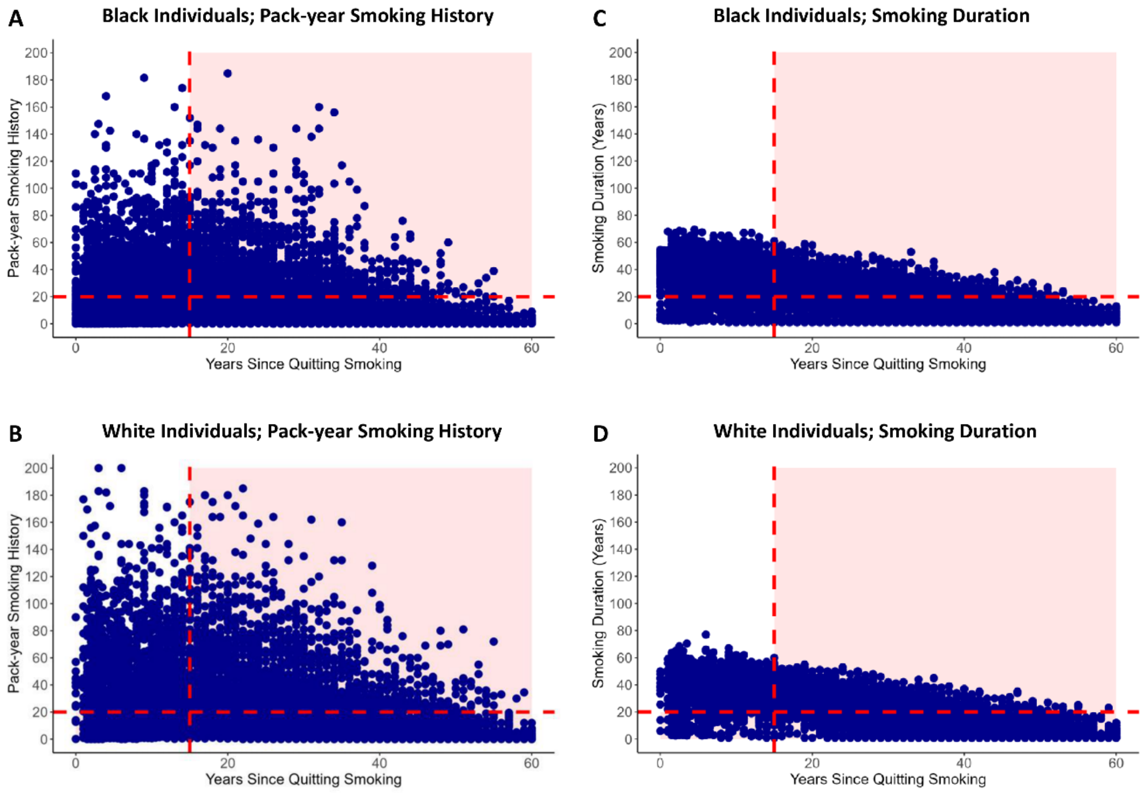

Supplementary Figure 4. Flow Diagram for Participants in the Black Women's Health Study (BWHS)

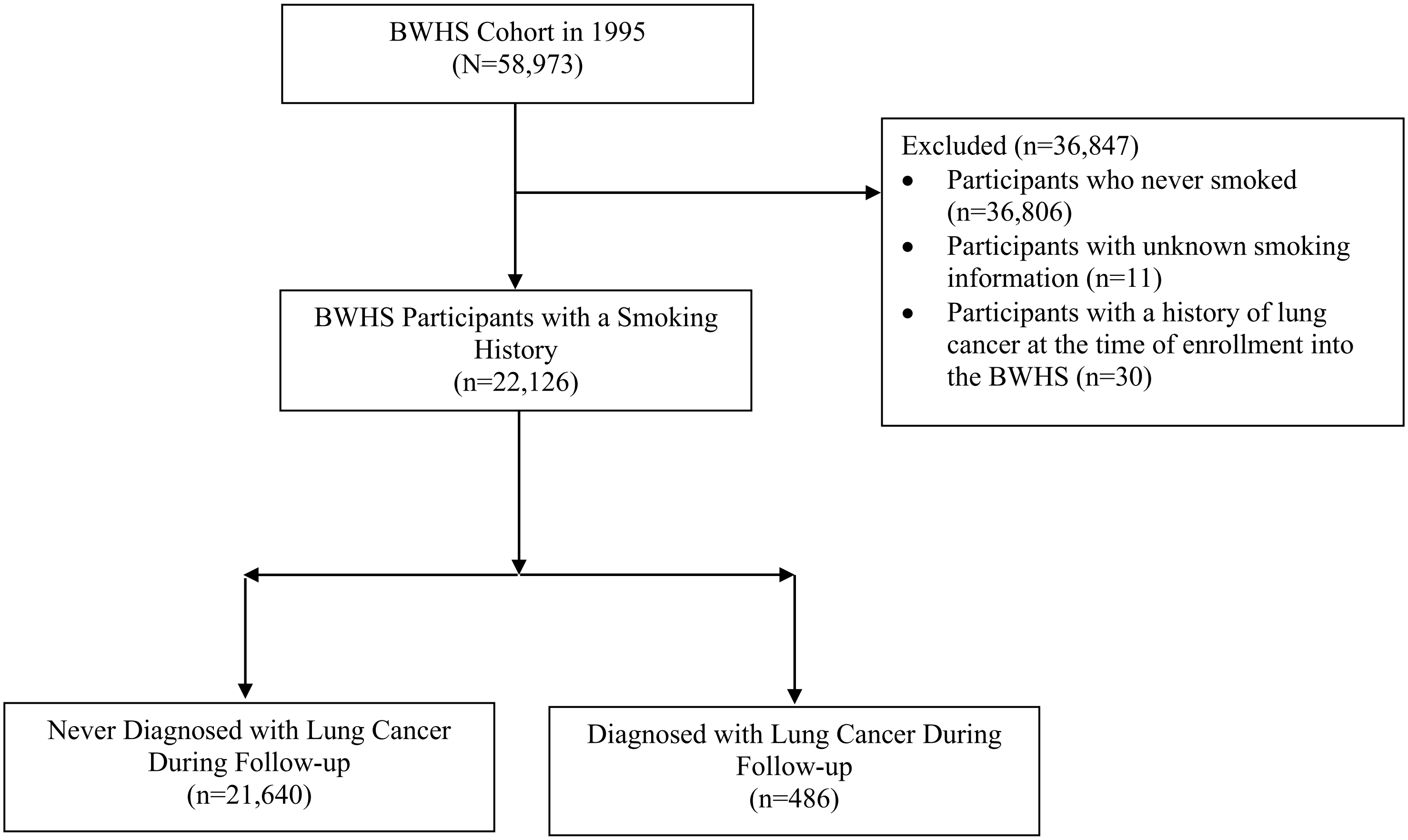

Supplemental Table 1. Characteristics of Participants in the Southern Community Cohort Study with Complete Versus Missing Smoking Information

Factor	Smoking Data Available	Missing Smoking Data	*P*-value
N	49703	2047	
Sex, n (%)			<0.001
Female	25660 (51.6)	1215 (59.4)	
Male	24043 (48.4)	832 (40.6)	
Race, n (%)			<0.001
White	16118 (32.4)	544 (26.6)	
Black	33585 (67.6)	1503 (73.4)	
Age at Last Follow-up, n (%)			<0.001
40-49 Years	1710 (3.4)	22 (1.1)	
50-59 Years	20492 (41.2)	543 (26.5)	
60-69 Years	18266 (36.8)	793 (38.7)	
70-79 Years	7826 (15.7)	553 (27.0)	
Over 80 Years	1409 (2.8)	136 (6.6)	
Comorbidity Index,[1] n (%)			<0.001
0	9290 (18.7)	268 (13.1)	
1	12273 (24.7)	448 (21.9)	
2	11656 (23.5)	475 (23.2)	
3+	15529 (31.2)	706 (34.5)	
Unknown	955 (1.9)	150 (7.3)	
Income, n (%)			<0.001
<$15,000	29806 (60.0)	981 (47.9)	
≥$15,000 and <$25,000	10108 (20.3)	373 (18.2)	
≥$25,000 and <$50,000	5933 (11.9)	315 (15.4)	
≥$50,000 and <$100,000	2580 (5.2)	219 (10.7)	
≥$100,000	735 (1.5)	76 (3.7)	
Unknown	541 (1.1)	83 (4.1)	
Highest Level of Education at Baseline			<0.001
Less than High School	15948 (32.1)	631 (30.8)	
High School	16963 (34.1)	575 (28.1)	
More than High School	16769 (33.7)	834 (40.7)	
Unknown	23 (<1)	7 (0.3)	
Self-reported Chronic Obstructive Pulmonary Disease at Baseline, n (%)			<0.001
No	44170 (88.9)	1879 (91.8)	
Yes	5456 (11.0)	162 (7.9)	

Unknown	77 (0.2)	6 (0.3)	
Family History of Lung Cancer, n (%)			0.005
No	42348 (85.2)	1698 (83.0)	
Yes	7355 (14.8)	349 (17.0)	
Incident Lung Cancer, n (%)			<0.001
No	47563 (95.7)	1990 (97.2)	
Yes	2140 (4.3)	57 (2.8)	

[1]Comorbidity index is based on the Charlson index, with modifications to account for information available on the Southern Community Cohort Study questionnaire.

Supplemental Table 2. Sensitivity and Specificity of the 2021 United States Preventive Services Task Force (USPSTF) Guideline vs. the Proposed Smoking Duration Guideline in the SCCS and BWHS

	**Black Individuals**		**White Individuals**	
**Sensitivity**	Percentage (95% CI)	n/N	Percentage (95% CI)	n/N
2021 USPSTF - SCCS	57.6 (55.0 to 60.3)	770/1,336	74.0 (71.0 to 77.0)	595/804
Proposed Guideline - SCCS	85.3 (83.4 to 87.2)	1,140/1,336	82.0 (79.3 to 84.6)	659/804
2021 USPSTF - BWHS	42.5 (38.1 to 47.1)	207/486	NA	NA
Proposed Guideline - BWHS	63.8 (59.3 to 68.1)	310/486	NA	NA
**Specificity**	Percentage (95% CI)	n/N	Percentage (95% CI)	n/N
2021 USPSTF - SCCS	63.6 (63.0 to 64.1)	20,498/32,249	49.7 (48.9 to 50.5)	7,610/15,314
Proposed Guideline - SCCS	29.3 (28.8 to 29.8)	9,456/32,249	38.5 (37.8 to 39.3)	5,903/15,314
2021 USPSTF - BWHS	86.8 (86.3 to 87.2)	18,784/21,643	NA	NA
Proposed Guideline - BWHS	71.7 (71.1 to 72.3)	15,509/21,643	NA	NA

Supplemental Table 3. Proportion of Individuals Diagnosed with Lung Cancer Who Would Have Qualified Under the 2021 United States Preventive Services Task Force (USPSTF) Guideline vs. the Proposed Smoking Duration Guideline in the SCCS Stratified by Race and Age Group

	**2021 USPSTF**		**Proposed Guideline**	
	Percentage (95% CI)	n/N	Percentage (95% CI)	n/N
Black				
Age 50-59	57.8 (53.4 to 62.2)	279/483	97.1 (95.6 to 98.6)	469/483
Age 60-69	68.0 (63.8 to 72.1)	331/487	93.4 (91.2 to 95.6)	455/487
Age 70-80	60.2 (54.3 to 66.0)	160/266	81.2 (76.5 to 85.9)	216/266
White				
Age 50-59	86.0 (81.1 to 90.9)	166/193	99.5 (98.5 to 100)	192/193
Age 60-69	86.2 (82.4 to 89.9)	280/325	92.0 (89.1 to 94.9)	299/325
Age 70-80	67.4 (61.2 to 73.6)	149/221	76.0 (70.4 to 81.6)	168/221

Supplemental Table 4. Sensitivity and Specificity of the Proposed Duration Guideline Using Different Smoking Duration Thresholds in the SCCS and BWHS

**Sensitivity, % (95% CI)**	**≥ 10 Years**	**≥ 20 Years**	**≥ 30 Years**	**≥ 40 Years**	**≥ 50 Years**
White - SCCS	82.0 (79.3 to 84.6)	82.0 (79.3 to 84.6)	80.2 (77.5 to 83.0)	65.7 (62.4 to 69.0)	30.0 (26.8 to 33.1)
Black - SCCS	85.8 (83.9 to 87.7)	85.3 (83.4 to 87.2)	82.0 (80.0 to 84.1)	60.5 (57.9 to 63.1)	20.4 (18.3 to 22.6)
Black - BWHS	66.9 (62.5 to 71.1)	63.8 (59.3 to 68.1)	50.6 (46.1 to 55.2)	17.7 (14.4 to 21.4)	1.4 (0.6 to 3.0)
**Specificity, % (95% CI)**	**≥ 10 Years**	**≥ 20 Years**	**≥ 30 Years**	**≥ 40 Years**	**≥ 50 Years**
White - SCCS	37.1 (36.3 to 37.9)	38.5 (37.8 to 39.3)	43.1 (42.3 to 43.9)	61.5 (60.7 to 62.3)	89.0 (88.5 to 89.5)
Black - SCCS	27.2 (26.7 to 27.7)	29.3 (28.8 to 29.8)	36.2 (35.7 to 36.7)	60.7 (60.2 to 61.2)	90.9 (90.6 to 91.2)
Black - BWHS	65.3 (64.7 to 65.9)	71.7 (71.1 to 72.3)	81.7 (81.2 to 82.2)	92.4 (92.0 to 92.7)	98.6 (98.5 to 98.8)

Supplemental Table 5. Proportion of Individuals, With or Without Lung Cancer, Who Would Have Qualified Under the 2021 United States Preventive Services Task Force (USPSTF) Guideline vs. the Proposed Smoking Duration Guideline in the SCCS and BWHS

	**Black Individuals**		**White Individuals**	
	Percentage (95% CI)	n/N	Percentage (95% CI)	n/N
2021 USPSTF - SCCS	37.3 (36.8 to 37.8)	12,521/33,585	51.5 (50.7 to 52.3)	8,299/16,118
Proposed Guideline - SCCS	71.3 (70.8 to 71.7)	23,933/33,585	62.5 (61.7 to 63.2)	10,070/16118
2021 USPSTF - BWHS	13.9 (13.4 to 14.3)	3,066/22,126	NA	NA
Proposed Guideline - BWHS	29.1 (28.5 to 29.7)	6,444/22,126	NA	NA

Supplemental Table 6. Characteristics of Southern Community Cohort Study (SCCS) Participants, With and Without Lung Cancer, Meeting the United States Preventive Services Task Force (USPSTF) 2021 Guideline vs. the Proposed Guideline Based on Smoking Duration Among All SCCS Participants, White SCCS Participants, and Black SCCS Participants

Factor	USPSTF 2021 Guideline – **All SCCS Participants**	Proposed Guideline – **All SCCS Participants**	USPSTF 2021 Guideline – **White SCCS Participants**	Proposed Guideline – **White SCCS Participants**	USPSTF 2021 Guideline – **Black SCCS Participants**	Proposed Guideline – **Black SCCS Participants**
N	20820	34003	8299	10070	12521	23933
Sex, n (%)						
Female	9774 (46.9)	16719 (49.2)	4652 (56.1)	5850 (58.1)	5122 (40.9)	10869 (45.4)
Male	11046 (53.1)	17284 (50.8)	3647 (43.9)	4220 (41.9)	7399 (59.1)	13064 (54.6)
Race, n (%)						
White	8299 (39.9)	10070 (29.6)	8299 (100.0)	10070 (100.0)	0 (0.0)	0 (0.0)
Black	12521 (60.1)	23933 (70.4)	0 (0.0)	0 (0.0)	12521 (100.0)	23933 (100.0)
Age at Last Follow-up, n (%)						
50-59 Years	9470 (45.5)	16989 (50.0)	3893 (46.9)	4930 (49.0)	5577 (44.5)	12059 (50.4)
60-69 Years	8584 (41.2)	13207 (38.8)	3142 (37.9)	3698 (36.7)	5442 (43.5)	9509 (39.7)
70-80 Years	2766 (13.3)	3807 (11.2)	1264 (15.2)	1442 (14.3)	1502 (12.0)	2365 (9.9)
Comorbidity Index,[1] n (%)						
0	3896 (18.7)	7125 (21.0)	1264 (15.2)	1601 (15.9)	2632 (21.0)	5524 (23.1)
1	5137 (24.7)	8706 (25.6)	1805 (21.7)	2232 (22.2)	3332 (26.6)	6474 (27.1)
2	4721 (22.7)	7596 (22.3)	1891 (22.8)	2263 (22.5)	2830 (22.6)	5333 (22.3)
3+	6660 (32.0)	9992 (29.4)	3162 (38.1)	3765 (37.4)	3498 (27.9)	6227 (26.0)
Unknown	406 (2.0)	584 (1.7)	177 (2.1)	209 (2.1)	229 (1.8)	375 (1.6)
Smoking Status, n (%)						
Formerly Smoked	4249 (20.4)	7932 (23.3)	1876 (22.6)	2492 (24.7)	2373 (19.0)	5440 (22.7)
Currently Smoked	16571 (79.6)	26071 (76.7)	6423 (77.4)	7578 (75.3)	10148 (81.0)	18493 (77.3)
Pack-year Smoking History, median (IQR)	39.0 (26.5, 52.0)	24.0 (13.5, 43.0)	45.0 (34.0, 63.0)	41.0 (24.0, 58.0)	34.8 (24.5, 46.0)	20.5 (11.3, 36.0)
Cigarettes per Day at Baseline,[2] median (IQR)	20.0 (12.0, 20.0)	10.0 (7.0, 20.0)	20.0 (20.0, 30.0)	20.0 (10.0, 30.0)	20.0 (10.0, 20.0)	10.0 (6.0, 20.0)
Smoking Duration, median (IQR)	43.0 (39.0, 48.0)	41.0 (36.0, 46.0)	43.0 (38.0, 48.5)	42.0 (37.0, 48.0)	43.0 (39.0, 48.0)	41.0 (36.0, 46.0)
Years Since Quitting Smoking, median (IQR)	8.5 (4.0, 12.0)	8.0 (4.5, 11.0)	8.5 (4.0, 12.0)	8.0 (4.5, 11.0)	8.5 (4.0, 12.0)	8.5 (4.5, 11.0)
Lung Cancer, n (%)						
No	19455 (93.4)	32204 (94.7)	7704 (92.8)	9411 (93.5)	11751 (93.9)	22793 (95.2)

Yes	1365 (6.6)	1799 (5.3)	595 (7.2)	659 (6.5)	770 (6.1)	1140 (4.8)

[1]Comorbidity index is based on the Charlson index, with modifications to account for information available on the Southern Community Cohort Study questionnaire.

[2]Data on cigarettes per day were obtained from the baseline questionnaire in the Southern Community Cohort Study. All other smoking variables were updated in accordance with follow-up data and reflect the smoking characteristics of participants at the time of lung cancer diagnosis (if they were diagnosed with lung cancer) or at the time of last follow-up (if they were never diagnosed with lung cancer).

Supplemental Table 7. Baseline Characteristics of Black Women's Health Study Participants Diagnosed with Lung Cancer

Characteristic	Black Women
N	486
Age at Diagnosis, median (IQR)	65.0 (58.0, 71.0)
Smoking Status at Diagnosis, n (%)	
Formerly Smoked	244 (50.2)
Currently Smoked	242 (49.8)
Pack-year Smoking History, median (IQR)	23.0 (13.5, 38.0)
Cigarettes per Day at Baseline,[1] median (IQR)	10.0 (5.0, 20.0)
Smoking Duration, median (IQR)	32.0 (25.0, 38.0)
Years Since Quitting Smoking, median (IQR)	13.5 (6.0, 21.0)
Highest Level of Education at Baseline, n (%)	
Less than High School	42 (8.6)
High School	132 (27.2)
More than High School	312 (64.2)
Geographic Region at Baseline, n (%)	
Northeast	169 (34.8)
South	102 (21.0)
Midwest	127 (26.1)
West	88 (18.1)
Quartiles of Neighborhood Socioeconomic Status in the Entire BWHS Population at Baseline, n (%)	
Q1	144 (29.6)
Q2	113 (23.3)
Q3	112 (23.0)
Q4	86 (17.7)
Missing	31 (6.4)
BMI at Baseline, median (IQR)	26.2 (23.3, 30.0)
Chronic Obstructive Pulmonary Disease at Baseline, n (%)	
No	481 (99.0)
Yes	5 (1.0)
Family History of Lung Cancer, n (%)	
No	412 (84.8)
Yes	74 (15.2)

[1]Data on cigarettes per day were obtained from the baseline questionnaire in the Black Women's Health Study. All other smoking variables were updated in accordance with follow-up data and reflect the smoking characteristics of participants at the time of lung cancer diagnosis.

REFERENCES

1. Signorello LB, Hargreaves MK, Blot WJ: The Southern Community Cohort Study: investigating health disparities. J Health Care Poor Underserved 21:26-37, 2010

2. Rosenberg L, Adams-Campbell L, Palmer JR: The Black Women's Health Study: a follow-up study for causes and preventions of illness. J Am Med Womens Assoc (1972) 50:56-8, 1995
